# A Tumor and Immune-Related Micro-RNA Signature Predicts Relapse-Free Survival of Melanoma Patients Treated with Ipilimumab

**DOI:** 10.3390/ijms24098167

**Published:** 2023-05-03

**Authors:** Iyad Kobeissi, Islam Eljilany, Tala Achkar, William A. LaFramboise, Lucas Santana-Santos, Ahmad A. Tarhini

**Affiliations:** 1 Cutaneous Oncology and Immunology Department, H. Lee Moffitt Cancer Center and Research Institute, Tampa, FL 33612, USA; 2 Hematology Department, University of Pittsburgh Medical Center, Pittsburgh, PA 15213, USA; 3 Pathology and Laboratory Medicine Department, Allegheny Cancer Institute, Allegheny Health Network, Pittsburgh, PA 15524, USA; 4 Pathology Department, Feinberg School of Medicine, Northwestern University, Chicago, IL 60611, USA; 5 Oncologic Sciences Department, Morsani College of Medicine, University of South Florida, Tampa, FL 33602, USA

**Keywords:** biomarkers, immunotherapy, melanoma, miRNA, microRNA

## Abstract

Despite the unprecedented advances in the treatment of melanoma with immunotherapy, there continues to be a major need for biomarkers of clinical benefits and immune resistance associated with immune checkpoint inhibitors; microRNA could play a vital role in these efforts. This study planned to identify differentially expressed miRNA molecules that may have prognostic value for clinical benefits. Patients with surgically operable regionally advanced melanoma were treated with neoadjuvant ipilimumab (10 mg/kg intravenously every 3 weeks × two doses) bracketing surgery. Tumor biospecimens were obtained at baseline and surgery, and microRNA (miRNA) expression profiling was performed on the tumor biopsies. We found that an expression profile consisting of a 4-miRNA signature was significantly associated with improved relapse-free survival (RFS). The signature consisted of biologically relevant molecules previously reported to have prognostic value in melanoma and other malignancies, including miR-34c, miR-711, miR-641, and miR-22. Functional annotation analysis of target genes for the 4-miRNA signature was significantly enriched for various cancer-related pathways, including cell proliferation regulation, apoptosis, the MAPK signaling pathway, and the positive regulation of T cell activation. Our results presented miRNAs as potential biomarkers that can guide the treatment of melanoma with immune checkpoint inhibitors. These findings warrant further investigation in relation to CTLA4 blockade and other immune checkpoint inhibitors. ClinicalTrials.gov NCT00972933.

## 1. Introduction

The incidence of melanoma over the past few decades has significantly evolved worldwide [1]. The American Cancer Society estimated the 2023 number of new cases of melanoma and melanoma-related deaths in the United States to be 97,610 and 7990 cases, respectively [2]. Despite having surgically resectable melanoma, some patients continue to be at high risk for disease relapse and death. This includes patients with stages IIB-C melanoma with a deep primary melanoma, with or without ulceration, and stages IIIA-D that are based on regional lymphatic metastases. These patients who present with clinically detectable locoregionally advanced disease may benefit from systemic therapy with immune checkpoint inhibitors (ICIs) either as adjuvant therapy or preoperative neoadjuvant therapy [3,4]. Neoadjuvant immunotherapy trials have demonstrated promising outcomes, including high rates of pathologic complete responses for patients with clinically detectable locoregionally advanced and operable melanoma. Currently, postoperative adjuvant systemic therapy is the primary standard of care in patients with high-risk surgically resectable melanoma [5]. For example, Ipilimumab 3 or 10 mg/kg (Yervoy^®^, Bristol-Myers Squibb, New York, NY, USA), which is a monoclonal antibody ICI that blocks Cytotoxic T lymphocyte antigen-4 (CTLA4), has demonstrated significant and durable clinical activity in the management of patients with metastatic melanoma [6]. Therefore, in 2011, Ipilimumab was approved as an adjuvant treatment for people who have cutaneous melanoma and have had a complete resection, including a total lymphadenectomy, but still have cancer in their lymph nodes [7]. Hence, the number of individuals eligible for adjuvant therapy treatment is likely to steeply rise in future years. This will be accompanied by a growing desire for a better prediction of those patients at high risk of recurrence in whom the intervention is expected to be beneficial, to avoid over-treating patients who are likely to have been cured of the disease through surgery alone [8].

MicroRNAs (miRNAs) are short non-coding RNA molecules that regulate target messenger RNAs (mRNAs) through the 3′-untranslated region (UTR) of target genes [9]. Previous studies have supported the essential role of miRNAs in the immunomodulation of patients with cancer because they can function as tumor suppressors or tumor inducers [10,11,12]. In comparison to their “normal” physiological counterparts, miRNA expression profiles in cancer cells are aberrant. Based on the genes they target, miRNAs have been demonstrated to either hinder tumor growth or promote it. Melanoma progression can be impacted by either an increase in oncogenic microRNAs (oncomiRs) due to miRNA gene amplification and translocation, or a decrease in anti-oncomiRs due to deletions, mutations, promoter methylation, or abnormal processing [13]. Some miRNAs are significantly upregulated in melanoma. For instance, miR-182 promotes metastasis by suppressing Forkhead Box O3 (FOXO3) and melanocyte-inducing transcription factor (MITF) [14]. While miR-214 upregulation in melanoma cell lines significantly promotes tumor cell migration, invasion, and resistance to chemotherapy in melanoma [15]. In addition, miR-21 is upregulated in cutaneous melanomas compared to benign nevi. MiR-21 correlates with acquiring a metastatic phenotype and a worse prognosis [16,17]. The expression of miR-222 in melanoma tissue was higher in patients who did not benefit from treatment with ipilimumab [18]. However, little has been reported regarding the role of miRNAs as biomarkers of clinical benefits associated with ICIs [19]. To our knowledge, the role of miRNAs as potential ICIs response predictors in melanoma remains largely unexamined. In this study, miRNA expression profiling analysis of patients treated with neoadjuvant ipilimumab was conducted with the intent to identify differentially expressed miRNA molecules that may have prognostic value in relation to clinical benefits with ICIs and provide additional insights into the underlying immune mechanisms with CTLA4 blockade.

## 2. Results

Demographics and baseline characteristics data on the 27 patients who were included in the analysis are presented in Table 1. As can be seen from the table, the median age of patients was 53 years old, and most of the patients were men (67%). Furthermore, 21 patients suffered from a disease recurrence after surgical treatment for melanoma. As expected, the majority of cases (*n* = 23) had cutaneous primaries and just above half of patients (55%) had a component of in-transit metastatic melanoma. The bottom half of the table shows the overall staging/classification at study entry for those newly diagnosed or as an estimated risk classification for those with the recurrent disease that was IIIB (3; N2b) or IIIC (24; N2c, N3), utilizing the AJCC v.7 staging criteria that were in effect during study conduct [20].

**Figure 1:**
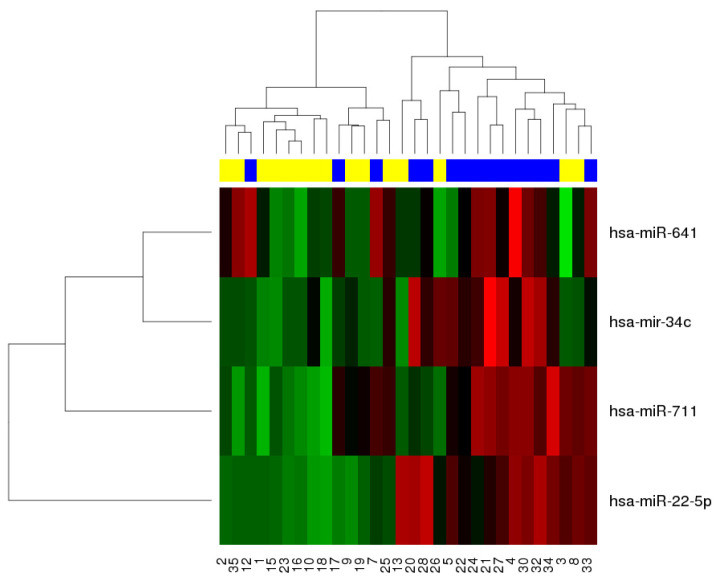
Biclustering analysis of differentially expressed genes by relapse-free survival (RFS). In the top bar, yellow represents samples of patients with RFS > 1 year, and blue represents those of patients with RFS of ≤1 year. In the body of the heat map, red shows up-regulated genes, and green shows down-regulated genes.

**Figure 2:**
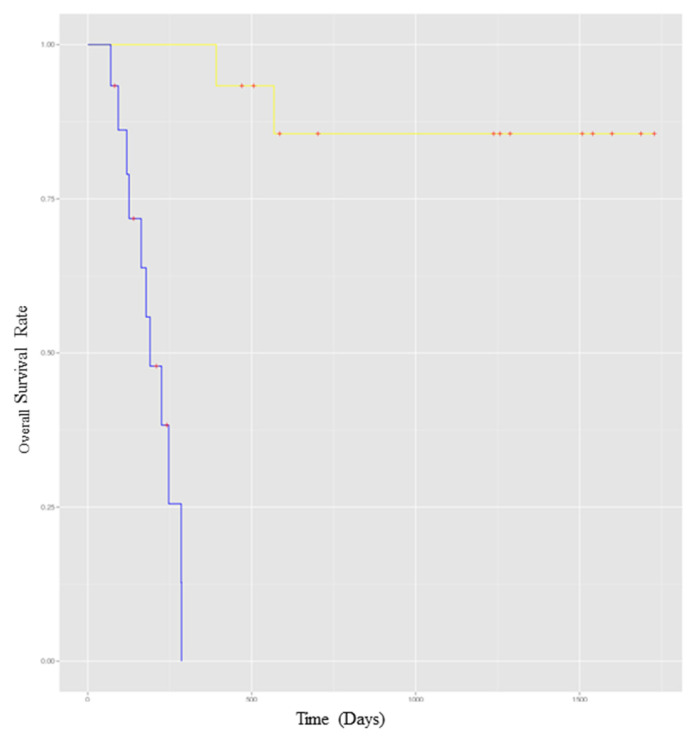
Time-to-event analysis comparing overall survival (OS) rate based on a 4-miRNA. Signature expression at baseline (before the initiation of neoadjuvant ipilimumab). The yellow line demonstrates patients with low expression of the 4-miRNA signature; the blue line demonstrates patients with high expression of the 4-miRNA signature. *Y*-axis represents the overall survival rate, and the *X*-axis represents the time in days.

As shown in Figure 1, the baseline expression profile of a 4-miRNA signature is associated with improved relapse-free survival (RFS). The four miRNA molecules were miR-34c, miR-711, miR-641, and miR-22. The full list containing all differentially expressed miRNAs, along with the statistical data such as fold change, *p*-value, and False Discovery Rate (FDR) is available in Supplementary Material (Table S1). Furthermore, time-to-event analysis was conducted using the 4-miRNA signature and the difference in RFS based on miRNA expression is presented in Figure 2. The figure shows that patients with an up-regulated 4-miRNA expression signature had a lower overall survival (OS) rate than patients with a down-regulated 4-miRNA expression signature. Furthermore, targets of the selected miRNAs were obtained from miRTarBase, where 212 genes targeted by the four miRNA molecules were identified. These genes are summarized in Supplementary Material (Table S2).

**Figure 3:**
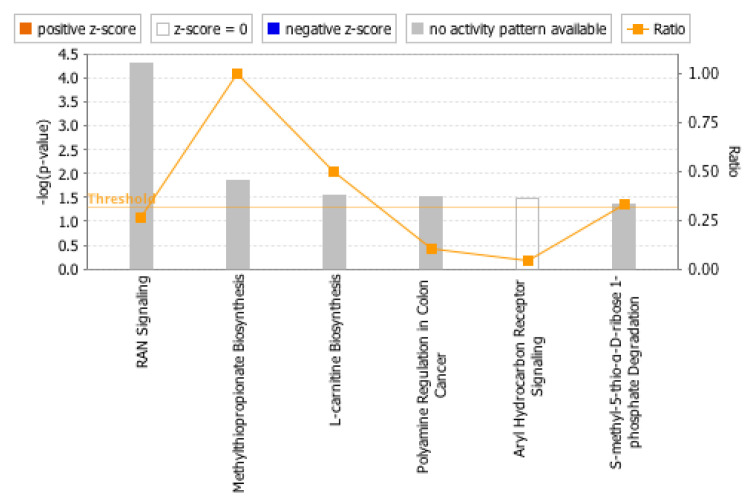
Canonical pathways as adapted from Ingenuity Pathway Analysis (IPA). IPA was used to determine the functional annotation of target genes for the 4-miRNA signature differentially expressed in patients treated with neoadjuvant ipilimumab based on the relapse-free survival outcomes. The threshold for significance is depicted by the horizontal orange line and the five pathways that achieve significance are indicated on the abscissa with the most significant values to the left. The ratio is the number of molecules in a given pathway that meet the cutoff criteria divided by the total number of molecules that make up the pathway in the reference data set. A ratio of 1 indicates that every molecule in the canonical pathway is a target of at least one of the miRNAs in the signature. The *p*-values in the −log(*p*-value) are from Fisher’s exact test that measures the significance of the overlap between analysis-ready genes in the dataset and genes within a pathway or function.

**Figure 4:**
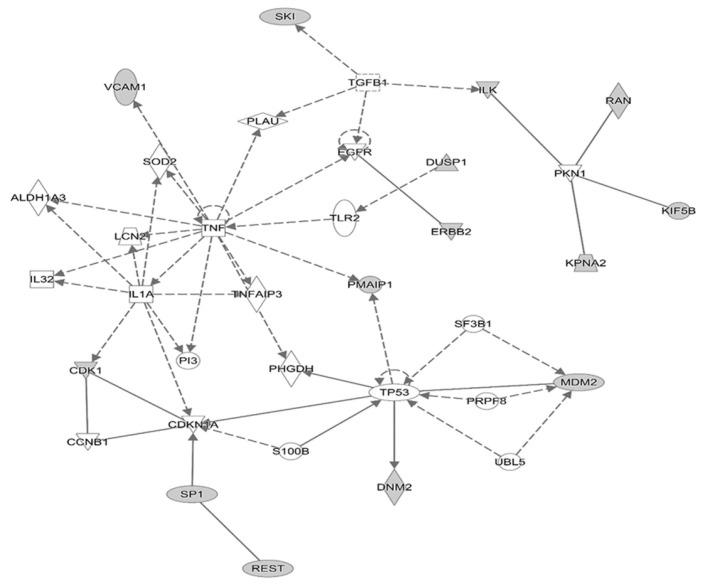
Cell death and survival, cell cycle, and cellular movement molecular network. This pathway represented the top canonical pathway. Adapted from Ingenuity Pathway Analysis (IPA).

The Ingenuity Pathway Analysis (IPA) identified pathways that were significantly associated with cancer-related and immune-related pathways. As expected, 139 out of 212 genes were functionally related to melanoma pathogenesis (*p* = 8.58× 10^−6^). Figure 3 indicates the downstream effects of the miRNA signature on target genes within the pathways. Identified pathways were significantly associated with the targets of the 4-miRNA signature; these included: RAN-related nuclear protein signaling (*p* = 5.16 × 10^−5^), methylthioproprionate biosynthesis (*p* = 1.45 × 10^−5^), L-carnitine biosynthesis (*p* = 2.88 × 10^−2^), polyamine regulation in colon cancer (*p* = 3.05 × 10^−2^), aryl hydrocarbon receptor signaling (*p* = 3.24 × 10^−2^). S-methyl-5-thio-D-ribose 1-phospate degradation is not statistically significant, hence, no *p*-value is shown for this. The IPA analysis presented in Figure 4 demonstrates that interleukin (IL)-1α, IL32, tumor necrosis factor (TNF), TNF alpha-induced protein 3 (TNFAIP3), and toll-like receptor 2 (TLR2) are among the immune-related genes involved in this pathway and identified as targets of the generated miRNA signature. As a ratio of one indicates that every molecule in the canonical pathway is a target of at least one of the miRNAs in the signature. That was the case for the methyl-propionate biosynthesis pathway, while half of the molecules in the L-carnitine biosynthesis pathway were activated by members of the miRNA signature. Curiously, the top canonical pathway involved cell death and survival, cell cycle, and cellular movement as the top associated network function.

## 3. Discussion

Advances in immune checkpoint targeted therapy opened the door for new transformative choices in cancer treatment. Immunotherapies using anti-CTLA4 and anti-PD1 monoclonal antibodies offer durable long-term survival benefits that were not previously achieved with traditional cytokine immunotherapy and chemotherapy in melanoma and other malignancies [21]. However, not all patients benefit from treatment, which makes biomarkers of disease prognostic or therapeutic predictive values an urgent need that may allow clinicians to refine personalized treatment options [8]. Here, an initial objective of the project was to find differentially expressed miRNA molecules that may have predictive value in relation to ipilimumab’s therapeutic benefits RFS after immunotherapy and definitive surgery. Towards achieving this, we collected tumor tissue at baseline before initiating neoadjuvant immunotherapy and after the resection; then, we completed RNA microarray studies on tissue biopsies.

The most important result was that a 4-miRNA signature (miR-34c, miR-711, miR-641, and miR-22) was significantly associated with RFS in patients treated with neoadjuvant ipilimumab for 6 weeks at 10 mg/kg followed by definitive surgical resection. The IPA indicated a significant association of the four miRNA molecules with regulating neoplastic pathways. These miRNAs have biologically significant functions. For example, miR34c is linked to the development of melanoma, especially evident in uveal melanoma, where miR34c regulates cell proliferation and migration. This could be explained by the transfection of miR-34b/c into uveal melanoma cells that drastically lowers cell growth by targeting c-Met, which in turn downregulates the AKT signaling pathway, inhibiting cell proliferation and migration. Moreover, miR-34b/c inhibits cell growth via the cell cycle proteins CDK4, CDK6, and Rb [22].

In fact, the interaction between the NKG2D receptor of natural killer (NK) cells with cytotoxic T lymphocytes (CTLs), and UL16 binding protein 2 (ULBP2), which is an NKG2D ligand expressed in tumor cells, allows NK cells to kill the tumor cells. Adding to that, miRNAs regulate NK cells’ function. For that reason, the overexpression of miR-34a and miR-34c in melanoma cells downregulates the ability of NK cells to recognize melanoma cells [23]. Not only that, but miRNAs also participate in the modulation of T-cell activity. For instance, the results of this study indicate antigen processing 1 (TAP1) and human leukocyte antigens (HLA-A) as molecular targets of our miRNA signature. Importantly, TAP1 and HLA-A are essential components of T cell function, and high expression of these molecules was found to increase the survival probability of melanoma patients [23]. Moreover, since miRNA influences PD-L1 expression, immunotherapy with high expression of miR34c therefore results in the downregulation of PD-L1 mRNA expression in breast cancer [24].

Undoubtedly, miRNA function is cell-type dependent, therefore, miR-34c may function as a tumor suppressor in triple-negative breast cancer (TNBC), and regulates TNBC invasiveness via the MAP3K2 pathway [25]. While in hepatocellular cancer (HCC), it functions as a tumor promoter and is overexpressed in HCC tissues, and is associated with poor survival [26]. In contrast, low miR-34c levels have been associated with the presence of lymph node invasion in head and neck squamous cell carcinoma [27].

Similarly, our findings demonstrated that high expression of miR-711 was associated with lower RFS. This finding aligns with an earlier study published in 2011 by Ralfkiaer and colleagues, who found that miR-711 was more expressed in patients with cutaneous T-cell lymphomas than in patients with benign dermatosis and normal skin [28]. This consistency was also disclosed in breast cancer patients, where it was found that higher miR-711 was significantly associated with poor OS [hazard ratio (HR), 2.549; 95% CI, 1.303–4.988; *p* = 0.006] and disease-free survival time (HR, 2.873; 95% CI, 1.392–5.929; *p* = 0.004) [29]. The main role of miR711, as explained in the same study, may be that miR-711 induces oncogenic proliferation and increases the migration and invasion of the cancer cell [29]. On the other hand, miR-711 regulates cell proliferation and has different roles reported in cancer tissues. For example, miR-711 was associated with cell cycle regulation in gastric cancer tissues. When upregulated, it arrests cell growth by downregulating the expression of cyclin-dependent kinases 4 (CDK4) [30]. These examples underline the importance of microRNAs as cancer-specific biomarkers, which can play different roles in different malignancies [31]. In this study, high expression of miR-641 was found to decrease RFS in patients with melanoma treated with ipilimumab. This result reflects those of Mueller et al. (2009), who also found that miR-641 was overexpressed in the early progression of melanocytes and primary melanoma cell lines [32]. Our result mirrors those obtained from an in-silico analysis that found that miR-641 was upstream in patients with cervical cancer. The study referred this observation to the fact that miR-641 might be responsible for the migration and invasion of Hela cells [33]. This finding is contrary to previous findings, which mentioned that mouse double minute 2 (MDM2) is an oncogene that acts as a negative regulator of p53, and it is overexpressed in a variety of human tumors, including melanoma. It has been reported that miR-641 overexpression is associated with decreasing expression levels of MDM2 and increasing p53 expression, which led to the inhibition of cell growth and the induction of cell apoptosis in human lung cancer cells [34]. Interestingly, alrizomadlin (APG-115), an MDM2 inhibitor, was recently studied in a phase II study in patients with unresectable or metastatic melanoma [35].

Perhaps one of the most obvious effects of the miRNAs in our analysis on the RFS was shown in miR-22, as the relation between the down- and up-regulated gene and the RFS was extremely apparent. As per our study outcomes, miR-22 negatively affected the survival rate. This result is supported by another research headed by Sand et al. in 2013, who revealed that, as well as being correlated with the progression of cutaneous malignant melanoma (CMMM) and primary PCMM, the upregulation of miR-22 was found to be expressed in cutaneous malignant melanoma metastases (CMMM) [36]. Several factors could explain this observation. Firstly, there exists a considerable body of evidence that miR-22 regulates cancer growth and epithelial-mesenchymal transition (EMT). Secondly, miR-22 affects cancer progression in various malignancies via distinct pathways. For example, miR-22 promotes breast cancer growth by suppressing acetylase TIP60, an epigenetic modifier and EMT promoter [37]. Furthermore, miR-22 is involved in hepatocellular carcinoma, which induces cell proliferation, migration, and invasion by downregulating CD147 [38]. Moreover, miR-22 seems to be involved in non-small cell lung cancer (NSCLC) [39]. Notwithstanding, miR-22 has been shown to suppress cervical and breast cancer cell proliferation by activating the pRb signaling pathway and p53 expression. In colorectal and renal cell carcinoma, miR-22 inhibits EMT and distant metastases [37]. The ambivalence regarding the role of miR-22 could be attributed to the fact that studies have shown that miRNAs can act both as tumor suppressors and oncogenes depending on their target genes [13].

With an in-depth look at the proto-oncogenes in our results, we find that the epidermal growth factor receptor (EGFR) is a molecular target, which is a transmembrane protein involved in the autocrine growth of melanoma cells, and the overexpression of EGFR has been reported to result in BRAFi/MEKi resistance [40]. We also found an association of the current study’s miRNA signature with ERBB2 (also known as HER2, neu, and NGL), which is known to be amplified in acral lentiginous melanoma and mucosal melanoma [41]. Interestingly, some of the target genes identified serve as biomarkers of survival in melanoma. For instance, S100B could serve as a strong baseline marker for survival in melanoma patients receiving anti-PD-1 immunotherapy [42]. In an analysis of a large cohort of patients with high-risk melanoma, Tarhini et al. reported that baseline S100b levels were significantly associated with RFS [HR 1.70 (95% CI, 1.21 to 1.92; *p* < 0.001)] and OS [HR 1.44 (95% CI, 1.06 to 1.95; *p* = 0.0210)], where higher values signified a worse prognosis [43]. Another relevant target identified was SKI protein, which is a transcriptional coregulator highly expressed in melanoma [44]. The experimental downregulation of SKI was known to inhibit melanoma cell growth in vitro and in vivo. Among the target molecules is (Toll-like receptors) TLR2, which engages in the recognition and activation of innate immunity. In a cohort of 54 melanoma patients receiving anti-PD-1 antibody monotherapy, non-responding patients had abundant expressions of immune gene markers such as *TNFAIP3* (encoding A20) and *TLR3* in tumors, which was associated with a dampened immune response [45]. Finally, the signature regulated pathways were involved in TNF signaling, which constitutes a potent immune escape mechanism in the context of a T-cell-inflamed tumor microenvironment, conferring resistance to anti-PD-1 [46].

The microarray methodology we employed incorporated multiplex QA/QC standards and both analytical and clinical controls to assure the accuracy of the assay and standardization of the expression values across all cohorts. The hybridization procedure comprehensively targeted transcripts spanning the transcriptome, albeit within a fixed dynamic range determined by the fluorescence laser scanner. It is important to note that this approach cannot detect variant transcripts containing base substitutions and InDels, as well as alternative splicing and fusion events that are identified through de novo RNA sequencing. Furthermore, the sensitivity of RNA sequencing is based on read depth and can achieve an order of magnitude higher sensitivity than microarrays. Consequently, RNA sequencing may detect rare variants and rearrangements that are outside of the scope of our technology. Additionally, the results of this study must be interpreted with caution because this miRNA signature has not been experimentally validated yet. Consequently, an appropriate future direction will be to use quantitative RT-PCR and Western blot to measure mRNA and protein expression, respectively, in patients with melanoma treated with ipilimumab. It will also be necessary to preclinically assess the mechanisms, toxicity, and potential therapeutic efficacy of this 4-miRNA signature, in vitro, using different techniques such as primary cell, cell culture, or cell lines.

## 4. Materials and Methods

### 4.1. Patients

This study gathered data from a previously published study on patients with locally advanced melanoma who were treated with neoadjuvant ipilimumab [47]. Briefly, ipilimumab was intravenously administered to thirty-five patients at a dose of 10 mg/kg every three weeks for up to two doses before decisive surgery and another two doses after recovery from surgery. Tumor samples were also collected before and within 6–8 weeks after the final resection. One patient who had been included in the original trial, but who did not provide proper consent, was excluded from this study. In addition, seven individuals lacked either sufficient tumor tissue or RNA quality for the RNA microarray analyses. The study’s Institutional Review Board ethical approval was obtained from the University of Pittsburgh and all patients participating in the study provided written informed consent.

### 4.2. Laboratory Methods

The profiling of gene expression was completed on tumor tissues from 27 patients before (baseline) and after (6–8 weeks) initiating systemic neoadjuvant therapy with ipilimumab. Primarily, FFPE tumor specimens were manually micro-dissected using an inverted microscope (Nikon Eclipse TE200) to collect at least 90% of tumor cells for RNA purification. Then, cells were scraped from 5-micron-thick unstained slices on slides paired with serially cut hematoxylin and eosin-stained tissues, including tumor regions delineated by a surgical pathologist (U.R.).

According to the protocol (Qiagen, Valencia, Santa Clarita, CA, USA), the extracted RNA was purified using a Qiagen miRNeasy FFPE kit with nuclease-free water as the solvent. After that, its inclusion in subsequent in vitro amplification (IVT) experiments was evaluated by spectrophotometric absorption ratio [260/280 > 1.8 (NanoDrop, Wilmington, DE, USA)] for the determination of RNA concentrations, and RNA Integrity Index (RIN) values were measured by microchip electrophoresis (Agilent Bioanalyzer 2100, Agilent Technologies, Santa Clara, CA, USA). The profiling of the tumor tissues’ miRNA expression for their association with immunotherapeutic benefits was performed using Affymetrix miRNA arrays V.4.; then, miRTarBase 6.0 “http://mirtarbase.mbc.nctu.edu.tw” (accessed on 20 November 2015) and TargetScan 7.0 “https://www.targetscan.org/vert_80/” (accessed on 10 December 2015) were used to obtain the targets of the selected miRNAs. Then, in order to analyze the list of miRNA target genes’ functional annotation, the Database for Annotation, Visualization, and Integrated Discovery (DAVID) 6.7 “https://david.ncifcrf.gov” (accessed on 24 December 2015) was used. Lastly, the IPA used the target genes of these miRNAs to perform the enrichment pathway analysis, in which the Benjamini and Hochberg approach was utilized to control multiple tests.

### 4.3. Statistical Methods

Significance Analysis of Microarrays (SAM) with 1000 permutations was carried out to detect the differential expression (DE) of each miRNA molecule while controlling for FDR; FDR was set at 5% (*p*-value 0.05) statistical significance to control for Type 1 errors arising from multiple tests. Furthermore, differential expression analysis was performed. The RFS and OS rate were the clinical endpoints assessed. Relapse-free survival (RFS) in our study was defined as the length of time without recurrence of melanoma or death following definitive surgery. Using hierarchical biclustering, miRNA expression between patient groups with RFS ≤ 1 and RFS > 1 year was compared.

## 5. Conclusions

This study identified a 4-miRNA signature, the expression levels of which at baseline were significantly associated with clinical benefits of neoadjuvant ipilimumab. Future studies will allow for the validation of these findings and integration with other biomarkers.

## Figures and Tables

**Table 1:** Patient Demographics and Baseline Disease Characteristics.

Variable	Patients (N = 27)
Age, years; Median (range)	53 (40–87)
Cutaneous primary, *n* (%)	23 (85)
Mucosal primary, *n* (%)	3 (11)
Unknown primary, *n* (%)	1 (3)
Gender, *n* (%)	
Female	9 (33)
Male	18 (67)
Performance status (ECOG), *n* (%)	
0	19 (70)
1	8 (30)
Recurrent disease after prior surgery, *n* (%)	21 (78)
Presence of in-transit metastases, *n* (%)	15 (55)
Estimated risk stage, *n* (%)	
IIIB	3 (11)
IIIC	24 (89)

## Data Availability

All results relevant to this study are included in the article and Supplementary Material. Data are available upon reasonable requests to the corresponding author.

## Supplementary Materials

The following supporting information can be downloaded at: https://www.mdpi.com/article/10.3390/ijms24098167/s1.
