# Supplementary material for: A Tumor and Immune-Related Micro-RNA Signature Predicts Relapse-Free Survival of Melanoma Patients Treated with Ipilimumab
Source: Int J Mol Sci. 2023 May 3;24(9):8167. doi: 10.3390/ijms24098167 (PMC10179521; doi:10.3390/ijms24098167)
Supplement: Supplementary file 1 [file ijms-24-08167-s001.zip › Supplementary material Table S2.pdf]

Table S2: miRNA signature target genes identified using miRTarBase

**miRNA signature target genes**					
ADAMTS20	DCAF7	KIF5B	OAZ2	SP1	VGLL4
ADI1	DDX3X	KLHL21	P2RY1	SP140L	VSIG2
ADSS	DEPDC1B	KLHL32	PARP15	SPRYD4	WDR81
AFF2	DHCR24	KPNA2	PCSK4	SRD5A1	WWC2
AGBL5	DIAPH2	KPNA6	PDE3A	SSTR3	XRRA1
AIP	DLC1	KRTAP4-9	PIGN	ST13	YAF2
ALDH9A1	DNM2	LDLR	PMAIP1	STAM	YTHDC1
ANK1	DNTTIP2	LIMCH1	PNMA3	STAMBP	ZNF106
ANKRD36	DUSP1	LIN54	POGZ	STARD8	ZNF385A
AP3B1	ECSIT	LMAN2	POLDIP2	STYK1	ZNF99
ARHGAP21	EIF2AK4	LMBR1	PPM1A	SULT1B1	ZNRF3
ARSJ	EIF4A2	LPP	PPP6C	SYAP1	CRISPLD2
ATP6V1G1	EPHA4	MAGEF1	PRDM6	SYNRG	CYB561A3
AXIN2	ERBB2	MAP1B	PSD4	TAS2R30	ID4
AZIN1	F2RL2	MAPK10	PTP4A1	TES	ILK
B3GALT5	FADS1	MB21D2	QSOX1	TET3	NCKAP1L
B4GALT5	FAM208A	MDM2	RAB3GAP1	THOC2	NLN
BCLAF1	FAM89A	MFSD9	RAB44	THSD7A	SLC35G2
C1orf52	FGD4	MOB1A	RAN	TMCO1	SLC41A2
C6orf48	FGFR1OP2	MRPL10	RCAN1	TMEM98	UBN2
CALM3	FRK	MTMR12	REST	TNFAIP1	UBXN2A
CAND1	GDE1	MTRNR2L10	RPL10	TNFSF9	CREBRF
CANX	GDNF	MTRNR2L11	RPN2	TNPO1	hsa-miR-711
CAPRIN1	GLTSCR2	MTRNR2L3	RPS24	TNPO3	NCAN
CCDC113	GOLT1B	MTRNR2L7	RPS4Y1	TNRC6C	SLC25A37
CCDC93	GPR26	MYL12B	SAR1A	TNS1	UBE2V2
CCNT2	GRPEL2	NBPF1	SDE2	TOB1	
CDH7	GXYLT1	NBPF10	SEC24A	TPI1	
CDK1	HIATL1	NBPF11	SEPT2	TRAK1	
CDKN2B	HIST1H2AH	NBPF12	SGCD	TRIM66	
CEBPB	HNRNPA1	NBPF14	SH3TC2	TRIP13	
CLCN5	HOXD1	NBPF15	SIKE1	TTC37	
CLDND1	hsa-miR-22-5p	NBPF20	SKI	TXNIP	
CPA4	hsa-miR-641	NBPF3	SLC25A33	UBE2Q1	
